# Metabolomic Differentiation of Malpighiaceae From Dry and Humid Tropics via UHPLC‐MS/MS and Chemometrics

**DOI:** 10.1002/ansa.70061

**Published:** 2026-03-06

**Authors:** Jaqueline Munise Guimarães da Silva, Rafael Felipe de Almeida, Maria Luiza Zeraik

**Affiliations:** ^1^ Laboratory of Phytochemistry and Biomolecules Department of Chemistry State University of Londrina (UEL) Londrina PR Brazil; ^2^ C.E. Moss Herbarium, School of Animal, Plant and Environment University of Witwatersrand Johannesburg South Africa

**Keywords:** brazilian biome, dry tropical areas, mass spectrometry, secondary metabolites

## Abstract

Various factors can trigger water stress in plants, particularly in those growing in dry tropical regions. To survive under such conditions, plants produce metabolites with adaptive functions. However, metabolomic data on the leaves of Malpighiaceae species cultivated in both dry and humid tropical areas of Brazil remain scarce. This study aimed to evaluate seasonal water stress in 10 species (from 7 genera) of Malpighiaceae inhabiting contrasting Brazilian biomes: Cerrado and Caatinga (dry areas), and Atlantic and Amazon Forests (humid areas). Metabolic profiles obtained by liquid chromatography coupled to mass spectrometry were compared using Variable Importance in Projection Scores from Partial Least Squares Discriminant Analysis. The results revealed a clear distinction between the leaf metabolites from dry versus humid environments. Cyanidin‐3‐*O*‐sambubioside (positive ionization mode) and 3,4‐di‐*O*‐galloylquinic acid (negative ionization mode) were identified as key discriminant compounds. Additionally, 15 previously unreported metabolites were annotated in the chromatographic profiles of Malpighiaceae leaves. This is the first study to demonstrate the influence of water availability on metabolomic synthesis across multiple species of Malpighiaceae. By integrating chromatographic and chemometric approaches, this study proposes a novel analytical strategy capable of revealing how environmental conditions shape metabolomic synthesis, thereby reinforcing its methodological relevance within analytical science.

## 1 Introduction

Secondary metabolites are chemical compounds responsible for various ecologically relevant roles in plants, such as defence against insects and sun protection [1]. Depending on the metabolite class, the metabolomic fraction qualifies the plant material for therapeutic, medicinal and other purposes [2, 3].

The environment and conditions in which a plant grows, such as water availability, light exposure, temperature and latitude, directly affect the biosynthesis and accumulation of secondary metabolites in plants [1, 4]. A change in a single factor, such as water stress, compromises metabolomic synthesis [4]. However, stating that water scarcity leads to an increase in the synthesis of all secondary metabolites is a misconception, as only some play adaptive roles and are consequently produced in more significant proportions in the leaves [4, 5]. Water stress can be induced in plant tissues by various environmental conditions, including the occurrence environment [6].

Dry and humid areas differ in terms of seasonal water stress. While humid tropical areas maintain relatively constant soil moisture throughout the year due to factors such as proximity to rivers and water recycling, dry tropical areas lack continuous access to this resource. Thus, plants growing in humid environments can continue to develop even during dry seasons, whereas those grown in dry regions may be significantly affected by water scarcity. The main changes resulting from this stress occur in the leaves, where there is more significant variation in the metabolomic fraction [6, 7].

During the cultivation of plants in dry areas, transcription factors and metabolite biosynthetic pathways are activated, including those for phenolic acids, flavonoids and anthocyanins [8]. These metabolites play an essential role in mitigating the effects of water stress [9]. The influence of the cultivation site on the synthesis of secondary metabolites has been reported in the leaves of various plants, such as the Asian *Eucommia ulmoides* (Eucommiaceae), the African *Elaeis guineensis* (Arecaceae) and the Malagasy *Kalanchoe pinnata* (Crassulaceae) [10, 11, 12]. However, there are no reports in the literature of metabolomic and comparative analyses at the occurrence environment level (dry and humid) for leaves of Malpighiaceae species.

The botanical family Malpighiaceae is globally distributed, with Brazil being one of the central countries where Malpighiaceae plants are concentrated. Of the 72 genera (ca 1500 species), 46 are found in Brazil, including 599 species, of which 368 are endemic [13, 14]. Considering the pronounced climatic, seasonal and hydrological heterogeneity that shapes the distribution of Malpighiaceae across Brazil [7], investigating this family is particularly relevant, as such environmental gradients directly modulate secondary‐metabolite biosynthetic pathways. As a result, Malpighiaceae represents a suitable model for assessing how contrasting water‐availability conditions influence chemical diversity, ecological function and the potential biological activity of plant metabolites.

Between 2004 and 2025, information on Malpighiaceae was expanded through various analyses, contributing to new classifications and groupings, with metabolite analysis being one of the most significant studies for this scientific advancement [14, 15, 16, 17]. In the botanical family Malpighiaceae, the combination of metabolomic analyses of leaf extracts and chemometric tools, such as Variable Importance in Projection (VIP) Score from Partial Least Squares Discriminant Analysis (PLS‐DA), has contributed to discriminating different species and identifying metabolites responsible for larvicidal activity [16, 17, 18, 19].

Considering the remarkable structural diversity of plant metabolites in Malpighiaceae [15, 17, 18, 20], including the occurrence of numerous isomers and a broad range of molecular masses, one of the major challenges in metabolomic investigations is the selection of analytical methodologies capable of detecting these compounds with both high sensitivity and high specificity. Furthermore, the unambiguous discrimination of metabolites sharing identical nominal or exact masses remains a critical bottleneck, particularly in complex plant matrices. Overcoming this limitation requires not only the application of high‐resolution mass spectrometry but also the integration of advanced multivariate statistical and chemometric approaches, which together enhance metabolite annotation reliability, improve structural elucidation and enable more accurate biological interpretation of the data.

Analytical approaches are essential for resolving the complex metabolomic architecture of Malpighiaceae, particularly when environmental variables modulate biosynthetic pathways. UHPLC‐MS/MS offers high‐resolution detection of structurally diverse metabolites, while chemometric modelling enhances discrimination and interpretation of metabolic shifts. However, no study has yet applied this integrative approach to evaluate water–stress‐driven metabolomic variation in this family, representing a clear analytical knowledge gap that the present work addresses.

Thus, in this study, the metabolomic profiles obtained by Ultra‐High Performance Liquid Chromatography coupled to Mass Spectrometry (UHPLC‐MS/MS) from ten species belonging to seven genera of the Malpighiaceae family were compared using VIP Score (PLS‐DA) to identify the discriminant metabolites synthesized in the leaves of species growing in dry and humid tropical areas of Brazil.

## 2 Material and Methods

### 2.1 Chemicals and Materials

The solvents used in this study were ethanol, formic acid and methanol (Sigma‐Aldrich, St. Louis, Missouri, United States of America), as well as acetonitrile and ultrapure water (Fisher Scientific, Fair Lawn, New Jersey, United States of America). The equipment used in the extraction stage was: Qiagen TissueLyzer II (Qiagen, Hilden, Germany), Labconco CentriVap rotary evaporator (Labconco Corporation, Kansas City, United States of America.

For the separation and detection of metabolites, an UltiMate 3000 UHPLC system (Thermo Scientific, Massachusetts, United States) equipped with a Kinetex C_18_ reverse‐phase UHPLC column (Phenomenex, Torrance, United States of America) was coupled to a Maxis Impact ESI‐Q‐TOF mass spectrometer (Bruker Daltonics, Germany). The hexakis 1*H*,1*H*,2*H*‐difluoroethoxypheosphazene (Synquest Laboratories, Alachua, United States of America) was used for mass correction in MS/MS.

### 2.2 Plant Material and Preparation of Extracts

A total of 30 adult leaf samples from ten species belonging to seven genera of the Malpighiaceae family, growing in different biomes of Brazil, were collected in the field between 2014 and 2018 (Table 1). The identification of each leaf was confirmed by the botanist R. F. de Almeida (C. E. Moss Herbarium, School of Animal, Plant and Environment, University of Witwatersrand, Johannesburg, South Africa). After collection, the leaves (Table 1) were dried at 40°C for 3 days. This drying protocol (40°C for 3 days) is sufficiently mild to preserve the phytoconstituents, including thermolabile compounds. Moreover, research on plants reports that drying at this temperature does not significantly reduce phenolic constituents or other heat‐sensitive bioactive metabolites [17, 21]. Subsequently, the material was stored in the HUEFS herbarium at 18°C under continuous air conditioning (24 h per day) until extract preparation.

**TABLE 1:** Information on the 30 analysed adult leaves samples of Malpighiaceae.

No.	Species	Abbreviation	Occurrence environment	Brazilian biome	Voucher number (herbarium)
1	*Niedenzuella multiglandulosa*	*N. multiglandulosa*	Dry	Cerrado	1199 (SP)
2	*Niedenzuella multiglandulosa*	*N. multiglandulosa*	Dry	Cerrado	5206 (CGMS)
3	*Niedenzuella multiglandulosa*	*N. multiglandulosa*	Dry	Cerrado	523 (SP)
4	*Niedenzuella poeppigiana*	*N. poeppigiana*	Humid	Atlantic Forest	490 (RB)
5	*Niedenzuella poeppigiana*	*N. poeppigiana*	Humid	Atlantic Forest	490 (RB)
6	*Niedenzuella poeppigiana*	*N. poeppigiana*	Humid	Atlantic Forest	490 (RB)
7	*Heteropterys intermedia*	*H. intermedia*	Humid	Atlantic Forest	491 (RB)
8	*Heteropterys intermedia*	*H. intermedia*	Humid	Atlantic Forest	504 (SP)
9	*Heteropterys intermedia*	*H. intermedia*	Humid	Atlantic Forest	501 (SP)
10	*Heteropterys leona*	*H. leona*	Humid	Amazon Forest	8651 (BHCB)
11	*Heteropterys leona*	*H. leona*	Humid	Amazon Forest	8577 (BHCB)
12	*Heteropterys leona*	*H. leona*	Humid	Amazon Forest	8597 (BHCB)
13	*Acmanthera latifolia*	*A. latifólia*	Humid	Amazon Forest	8575 (CEPEC)
14	*Acmanthera latifólia*	*A. latifólia*	Humid	Amazon Forest	8580 (CEPEC)
15	*Acmanthera latifólia*	*A. latifolia*	Humid	Amazon Forest	8649 (CEPEC)
16	*Byrsonima coccolobifolia*	*By. coccolobifolia*	Dry	Cerrado	M2 R1943 (IAC 55280)
17	*Byrsonima coccolobifolia*	*By. coccolobifolia*	Dry	Cerrado	M3 R1943 (IAC 55280)
18	*Byrsonima coccolobifolia*	*By. coccolobifolia*	Dry	Cerrado	M6 R1944 (IAC 55278)
19	*Diplopterys pubipetala*	*D. pubipetala*	Dry	Cerrado	1126 (SP)
20	*Diplopterys pubipetala*	*D. pubipetala*	Dry	Cerrado	1334 (SP)
21	*Diplopterys pubipetala*	*D. pubipetala*	Dry	Cerrado	1030 (BHCB)
22	*Stigmaphyllon saxicola*	*S. saxicola*	Humid	Atlantic Forest	198029 (HUEFS)
23	*Stigmaphyllon saxicola*	*S. saxicola*	Humid	Atlantic Forest	551 (HUEFS)
24	*Stigmaphyllon saxicola*	*S. saxicola*	Humid	Atlantic Forest	580 (HUEFS)
25	*Banisteriopsis laevifolia*	*B. laevifolia*	Dry	Cerrado	1275 (SP)
26	*Banisteriopsis laevifolia*	*B. laevifolia*	Dry	Cerrado	1359 (SP)
27	*Banisteriopsis laevifolia*	*B. laevifolia*	Dry	Cerrado	3814 (RB)
28	*Banisteriopsis malifolia*	*B. malifolia*	Dry	Caatinga	54704 (IAC)
29	*Banisteriopsis malifolia*	*B. malifolia*	Dry	Caatinga	1344 (SP)
30	*Banisteriopsis malifolia*	*B. malifolia*	Dry	Caatinga	25 (BHCB)

To ensure sample homogeneity and representativeness, all plant materials (Table 1) were finely ground until a uniform powder was obtained, minimizing particle‐size variability and avoiding inhomogeneity, particularly in leaf tissues.

The leaves of the Malpighiaceae species listed in Table 1 were separately weighed (20 mg) and extracted with 1 mL of ethanol and water in a proportion 4:1 (v/v). Ethanol and water are suitable solvents for metabolite extraction in Malpighiaceae plants [16, 21]. The selected mass‐to‐solvent ratio (20 mg:1 mL) follows established UHPLC‐MS/MS metabolomic protocols, which recommend maintaining an excess of solvent to improve metabolite solubilization and prevent saturation. [17, 19, 21]. This ratio also supports efficient mixing during vortexing and sonication, improving extraction reproducibility [16, 19]. Thus, the adopted proportion yields representative extracts without compromising analytical performance.

The extraction procedure adopted in this study consisted of agitating the plant material with the extraction solvent under controlled agitation using a mechanical shaker [17, 21]. Initially, the plant material and the EtOH:H_2_O mixture (4:1, v/v) were stirred using a Qiagen TissueLyzer II for 5 min at 25 MHz. Subsequently, the mixture was homogenized again for 30 min. In sequence, the mixture was centrifuged for 15 min, and 300 µL of the supernatant was transferred to a deep‐well 96‐well microplate. The solvent was evaporated using a Labconco CentriVap rotary evaporator. Finally, the plates were sealed and stored at −80°C until chromatographic analysis.

### 2.3 Chromatographic Analysis by UHPLC‐MS/MS

For chromatographic analysis, the leaf extracts were suspended in 200 µL of methanol:water (4:1 v/v) and 2 µmol L^−1^ of sulfamethoxypyridazine (internal standard). This internal standard is used for monitoring the injections, such as retention time [21]. Chromatographic analyses were performed on a UHPLC system, using a C_18_ reverse phase UHPLC column of 1.7 µm × 50 × 2.1 mm. A Q‐TOF mass spectrometer with an electrospray ionization (ESI) source was used as the mass analyser.

The pumping system used water (A) and acetonitrile (B), both acidified with 0.1% formic acid (v/v), with a flow rate of 0.5 mL min^−^^1^ and an injection volume of 5 µL. The method employed was 5% Solvent B for 1 min, followed by a gradient from 5% to 100% in 5 min. The column was washed with 100% Solvent B for 2 min to enhance separation, then returned to the initial 5% in 1 min, reaching equilibrium for 1 min at 5% Solvent B.

The chromatographic gradient applied in this study has been previously tested and validated for UHPLC‐MS/MS analyses in different Malpighiaceae species, demonstrating robust performance and broad metabolite coverage for this plant family. This pre‐established method enables the detection of both early‐eluting hydrophilic compounds and more hydrophobic metabolites eluting under high‐organic conditions, ensuring adequate separation within the analytical window. Accordingly, this gradient was adopted as a reliable and established protocol for metabolomic profiling of Malpighiaceae tissues [17, 21].

The mass range analysed was 50–2000 Da in both positive and negative ionization modes. The spectrum acquisition rate was three spectra per second. Nitrogen gas (2 bar) was used as the nebulizer, with a capillary voltage of 4200 V, source temperature of 200°C and gas flow rate of 9 L min^−^^1^. For each spectrum, the five most intense ions were selected and fragmented using a collision‐induced dissociation energy ramp (22–50 eV).

After UHPLC‐MS/MS analyses, the results (chromatographic profiles in both positive and negative ionization modes) were individually processed using the MZmine software (version 2.53). For each ionization mode (positive and negative), the baseline and signal‐to‐noise ratio were adjusted separately. A mixture of six standards was used as a reference to evaluate the obtained spectra, with retention time and mass‐to‐charge (*m/z)* ions being the evaluation parameters. The processing workflow in MZmine involved the following steps: mass detection (centroid mode, thresholds of 1.0 × 10^3^ for MS^1^ and 1.0 × 10^1^ for MS^2^); chromatogram builder (minimum time span of 0.01 min, minimum height of 3.0 × 10^3^ and *m*/*z* tolerance of 20 ppm); chromatogram deconvolution (baseline cut‐off algorithm with minimum peak height of 1.0 × 10^3^, peak duration of 0.01–3 min and baseline level of 1.03), using median *m*/*z* centre calculation; *m/z* range for MS^2^ scan pairing set to 0.02 Da and retention‐time window to 0.1 min; isotope peaks grouper (*m/z* tolerance of 20 ppm, retention time tolerance of 0.1 min, maximum charge of 3 and the most intense isotope selected as representative); and join alignment (*m/z* tolerance of 20 ppm, weight for *m*/*z* and RT of 75 and 25, respectively, and tolerance of 0.1 min).

Thus, two internal performance parameters were monitored in every run— a retention‐time internal standard (sulfamethoxypyridazine) and a mass calibrant (hexakis(1*H*,1*H*,2*H*‐difluoroethoxy)phosphazene)—which enabled verification of chromatographic resolution and mass accuracy across all injections. While periodic pooled‐QC injections are considered best practice for monitoring long‐term instrumental drift, the combined use of these internal controls ensured stable analytical performance throughout data acquisition. Moreover, all raw chromatograms are publicly available on MassIVE, allowing independent confirmation of peak stability, retention‐time consistency and overall data quality.

The original chromatographic profiles obtained by UHPLC‐MS/MS (positive and negative ionization mode) are available on the MassIVE platform (MSV000085119).

### 2.4 Chemometric Analysis by VIP Score (PLS‐DA)

To identify the metabolites that discriminate the leaves of different Malpighiaceae species at the occurrence environment level (Table 1), the chromatographic profiles obtained by UHPLC‐MS/MS of the hydroethanolic leaf extracts were compared using PLS‐DA, specifically VIP Score. VIP Score analysis (PLS‐DA) was applied exclusively to identify discriminant metabolites. Therefore, the VIP Score and relative intensities of each metabolite were the parameters analysed for discrimination.

Two matrices, one for each ionization mode (positive and negative), were individually submitted to Metaboanalyst (version 6.0), where PLS‐DA was applied with 95% confidence for automatic clustering formation.

Each column of the matrix represented a sample (Table 1), and each row compiled the relative intensities of each sample according to the *m/z* values and their respective retention time (in ascending order). The matrices were preprocessed in Metaboanalyst, where the data were normalized by sum, log‐transformed (base 10) and auto‐scaled. These parameters were adopted from previous analyses selected based on the best separation trends.

To validate the VIP Score model PLS‐DA (assess metabolomic changes in leaves cultivated in both dry and humid tropical areas), the Q2 and R2 parameters obtained through cross‐validation (CV) were provided by MetaboAnalyst for each ionization mode analysed by UHPLC‐MS/MS. Q^2^ estimates the predictive ability of the model and is calculated via CV, in which predicted data are compared to the original data to compute the sum of squared errors. This prediction error, known as the Predicted Residual Sum of Squares (PRESS), is summed across all samples. The PRESS is then divided by the total sum of squares and subtracted from 1 to produce a value on the same scale as *R* ^2^. Accurate predictions are indicated by low PRESS values or high *Q* ^2^ values, ideally approaching 1.0.

### 2.5 Annotation of Metabolites in Chromatographic Profiles by UHPLC‐MS/MS

The annotation of metabolites in the profiles obtained by UHPLC‐MS/MS of the leaves of 10 species (Malpighiaceae) was done by using spectral libraries such as NIST, Massbank and Spectral Match. Thus, metabolomic annotation in the UHPLC‐MS/MS profiles was classified as Level 2b [22], that is, based on the identification of the molecular formula and MS^2^ fragments using computational tools, particularly online spectral libraries (NIST, Massbank and Spectral Match).

The combined use of these spectral libraries was adopted to increase the breadth of annotated compounds, thereby improving both the depth and reliability of the metabolite annotation process. These libraries were used for two purposes: the comparison between experimental spectra and spectra publicly available on the platform, and the confirmation of information such as chemical structure and precursor ion.

For both MS^1^ and MS^2^ ions, mass values were processed with four decimal places, a precision threshold that minimizes rounding errors and strengthens the confidence of the subsequent annotation workflow. Additionally, the exact mass value of the molecular ions from the metabolites was calculated in Exact Mass, and the error (ppm) was estimated for each annotation. The confidence level of the annotated compounds was determined solely based on MS/MS data [23].

## 3 Results and Discussion

**FIGURE 1:**
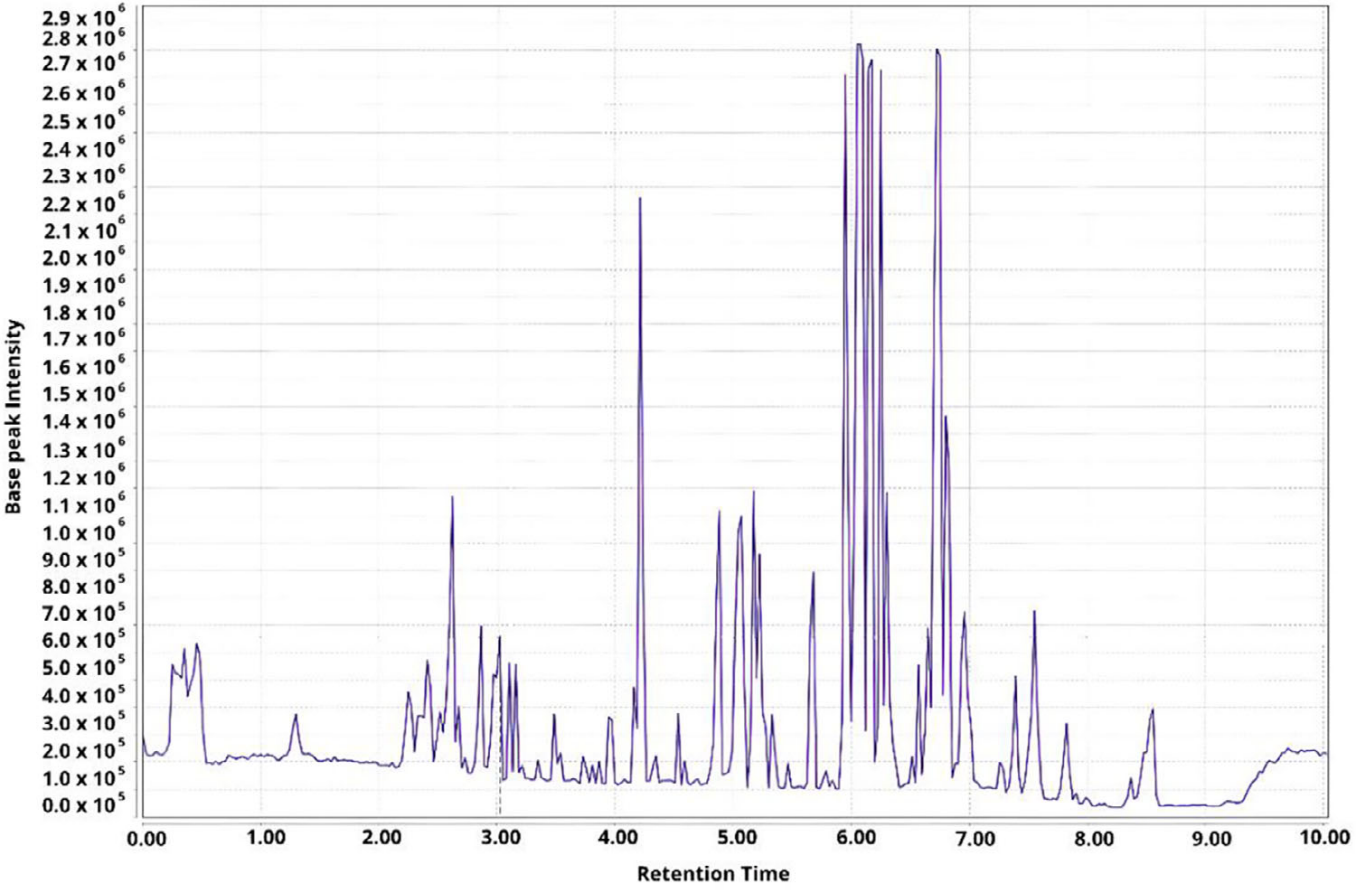
Chromatogram obtained by UHPLC‐ESI‐MS/MS (positive ionization mode) of the leaf extract of *Stigmaphyllon saxicola* (Sample 22—Table 1) prepared from extraction with ethanol:water (4:1 v/v).

The chromatographic profiles of Malpighiaceae leaves obtained by UHPLC‐MS/MS (as shown in Figure 1) were compared using VIP Scores (PLS‐DA) to identify the distinguishing metabolites specific to each occurrence environment and to assess the influence of dry and humid tropical areas. The comparison of chromatograms from the leaves of ten Malpighiaceae species, analysed in both positive and negative ionization modes, revealed metabolomic differences between leaf extracts from different environmental conditions. These differences are evident in the Score plots and VIP Scores for both ionization modes.

The integration of UHPLC–MS/MS chromatographic profiles with chemometric discrimination through VIP‐based PLS‐DA provides a high‐resolution analytical route for exploring secondary metabolite composition. This combined approach enhances feature detection, improves metabolite discrimination capacity and enables a more precise interpretation of metabolic variation between contrasting environments. By coupling advanced chromatographic data with multivariate statistical modelling, the present work establishes a robust analytical framework that strengthens methodological innovation in analytical science and expands the reliability of metabolomic differentiation in complex biological matrices.

### 3.1 Score Plot and VIP Score (PLS‐DA) Based on UHPLC‐MS/MS Profiles in Positive Ionization Mode

**FIGURE 2:**
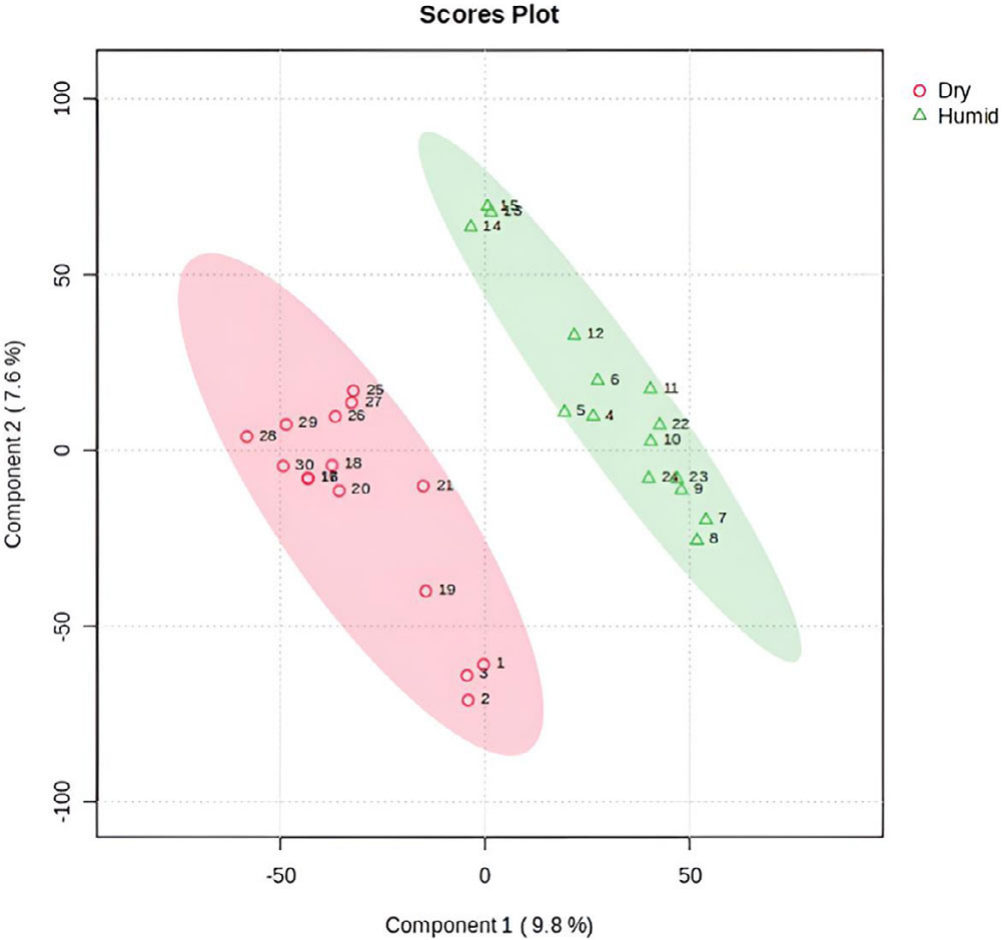
Score plot from PLS‐DA based on UHPLC‐MS/MS data (positive ionization mode), showing the separation of Malpighiaceae leaf samples from dry and humid tropical areas according to their metabolomic profiles.

The Score plot (PLS‐DA), generated from UHPLC‐MS/MS profiles in positive ionization mode, confirmed the discrimination of secondary metabolites synthesized by Malpighiaceae species cultivated in dry and humid tropical areas (Figure 2). The points (triangles) representing leaves from humid environments, whether from the Atlantic or Amazon Forests, clustered, with 95% confidence, in the positive region of Component 1. In contrast, the points (circles) corresponding to leaves from dry tropical areas (Cerrado and Caatinga) were positioned in the negative region of the same component. These results indicate that, despite the samples belonging to different genera and species of Malpighiaceae (Table 1), seasonal water stress plays a key role in modulating the synthesis of specific secondary metabolites that may contribute to plant adaptation and survival under drought conditions.

Since metabolomic synthesis is influenced by environmental factors [1, 4], the VIP Score from PLS‐DA was used to identify the metabolites responsible for differentiating plant samples from dry and humid tropical areas. In this analysis, two key measures are used: the VIP and the weighted sum of absolute regression coefficients. Each metabolite was assigned a Score based on its association with the environmental variable (dry or humid conditions). The coloured boxes on the right side of the VIP Score plots represent the relative abundance of each metabolite in the respective groups. Thus, higher VIP Scores indicate greater relevance for group discrimination. Metabolites with VIP values greater than 1.9 were considered the most significant for distinguishing between environmental conditions [24, 25].

To refine the selection of discriminant metabolites, we applied a VIP threshold of VIP ≥ 1.9. Although VIP ≥ 1.0 is considered the minimum relevance criterion, several metabolomics studies have adopted stricter cutoffs (typically 1.5–2.0) to ensure the retention of only the most influential variables and to minimize false‐positive contributions.[26, 27] This higher threshold was justified by the distribution of VIP values observed in our dataset, where the most discriminant features occurred between 1.9 and 2.1, supporting the robustness of the selected cutoff and its suitability for interpretation.

**FIGURE 3:**
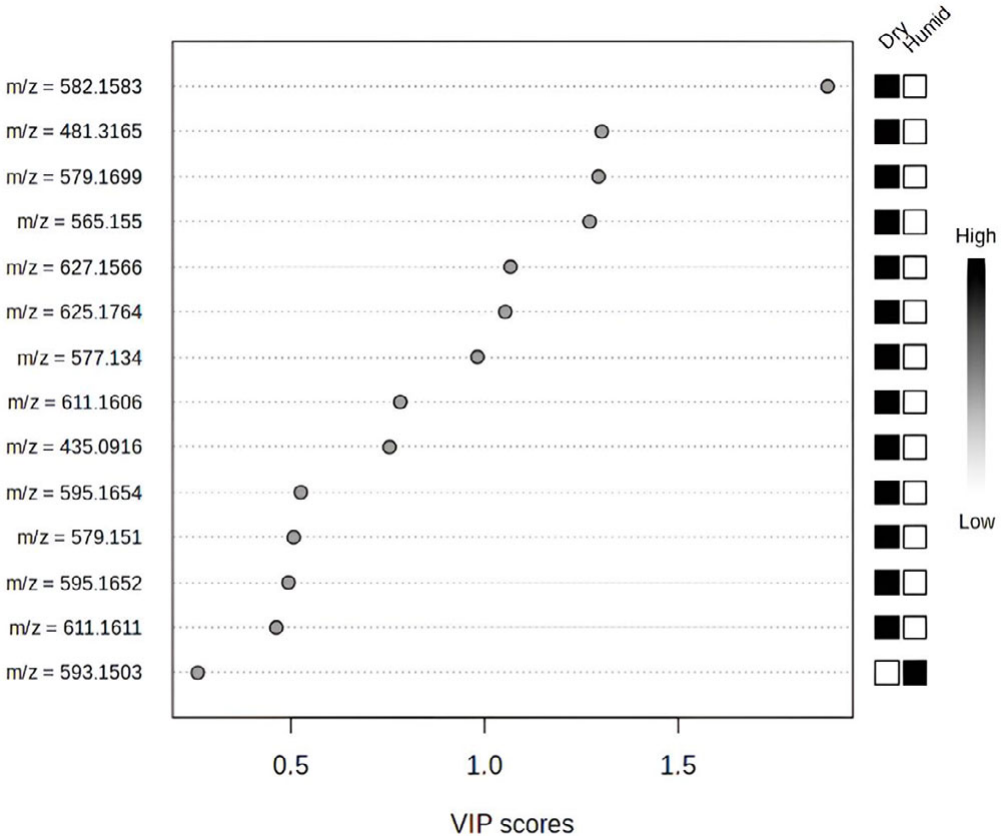
Variable Importance in Projection (VIP) scores from PLS‐DA based on UHPLC‐MS/MS data (positive ionization mode), showing the metabolites that best discriminate Malpighiaceae leaf samples from dry and humid tropical areas. Higher scores indicate greater contribution to group separation.

In total, 14 metabolites (detected in positive ionization mode) showed VIP Scores, of which 13 exhibited higher relative intensity in leaves from dry tropical areas (Figure 3). Table S1 summarizes the identified metabolites highlighted in the VIP Score analysis (Figure 3), along with the respective leaf samples in which they were detected.

The highest VIP Score was attributed to cyanidin‐3‐*O*‐sambubioside (Compound 7—Table S1), an anthocyanin‐class metabolite. Samples 28, 29 and 30, corresponding to *Banisteriopsis malifolia* leaves grown in the Caatinga biome, exhibited the most negative values in Component 1 (Figure 3) and the highest intensities of cyanidin‐3‐*O*‐sambubioside. Other leaf samples—*Niedenzuella multiglandulosa*, *Byrsonima coccolobifolia*, *Diplopterys pubipetala* and *Banisteriopsis laevifolia*—all cultivated in dry areas, also presented the molecular ion of this anthocyanin (Table S1).

Anthocyanins are pigments synthesized in plant tissues during specific growth stages or in response to environmental stimuli such as visible radiation and water stress [6, 28]. Under drought conditions, water loss from plant cells can lead to dehydration and even plasmolysis, which in turn stimulates the production and accumulation of anthocyanins, particularly in the leaves, but also in stems and roots [6, 28]. These compounds contribute to improved drought tolerance by lowering the osmotic potential, enhancing water uptake, and reducing transpiration losses. In addition to these physiological roles, anthocyanins also offer photoprotection and antioxidant activity, enabling plants to tolerate suboptimal water availability [6]. Thus, the cyanidin‐3‐*O*‐sambubioside is a key anthocyanin derived from the phenylpropanoid pathway, whose upregulation is widely associated with enhanced antioxidant defences required to counteract drought‐induced oxidative stress.

It is estimated that anthocyanin concentrations in leaves from dry environments are up to four times higher than in those from non‐stressed conditions [6, 28]. This reinforces the role of cyanidin‐3‐*O*‐sambubioside as a key discriminating compound between dry and humid tropical areas. While this metabolite had the highest importance in the VIP Score (Figure 3), other compounds such as the steroid ecdysterone (14) and the flavonoids vitexin‐2‐*O*‐rhamnoside (5) and isovitexin‐2‐*O*‐arabinoside (4) also contributed to the differentiation, though with lower VIP values. These metabolites are likewise associated with adaptive responses that enhance plant vitality in dry environments.

In addition to supporting the chemometric findings, the UHPLC‐MS/MS profiles (positive ionization mode) revealed the presence of previously unreported metabolites in the studied species. Six compounds were annotated for the first time in these Malpighiaceae species: isovitexin‐2‐*O*‐arabinoside (4), vitexin‐2‐*O*‐rhamnoside (5), delphinidin‐3‐*O*‐6‐*O*‐α‐rhamnopyranosyl‐β‐glucopyranoside (6), cyanidin‐3‐*O*‐sambubioside (7), kaempferol‐*O*‐pentosyl‐pentoside acetate (10) and genistein‐di‐*C*‐hexoside (11). The corresponding species in which these compounds were identified are listed in Table S1.

### 3.2 Score Plot and VIP Score (PLS‐DA) From Metabolomic Profiles by UHPLC‐MS/MS in Negative Ionization Mode

**FIGURE 4:**
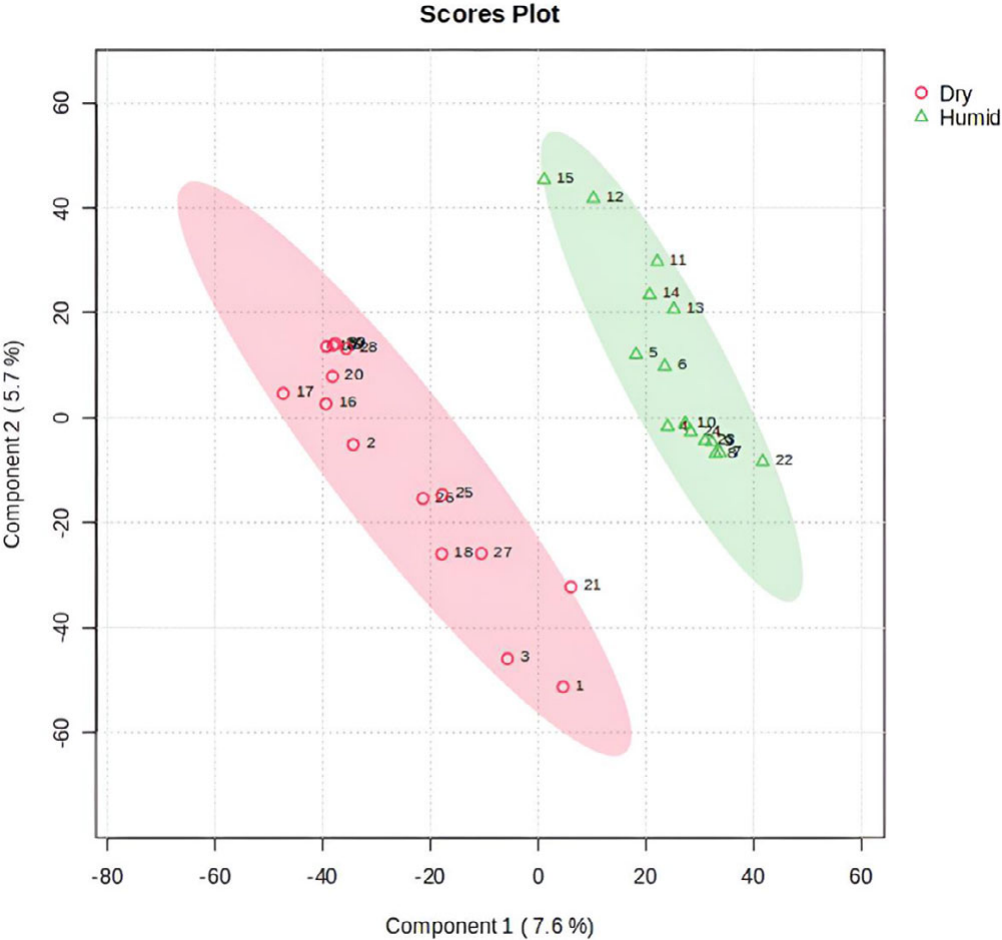
Score plot from PLS‐DA based on UHPLC‐MS/MS data (negative ionization mode), showing the separation of 30 Malpighiaceae leaf samples from dry and humid tropical areas according to their metabolomic profiles.

Chemometric analysis of the Score plot (PLS‐DA), based on chromatographic profiles obtained by UHPLC‐MS/MS in negative ionization mode, also revealed clear discrimination among the investigated plant samples (Figure 4). Scores located in the positive region of Component 1 (triangles) correspond to leaf samples cultivated in humid tropical areas, including the Atlantic and Amazon Forests. In contrast, points representing leaves from dry tropical areas, specifically the Cerrado and Caatinga biomes, are symbolized by circles and clustered in the negative region of Component 1 (Figure 4).

**FIGURE 5:**
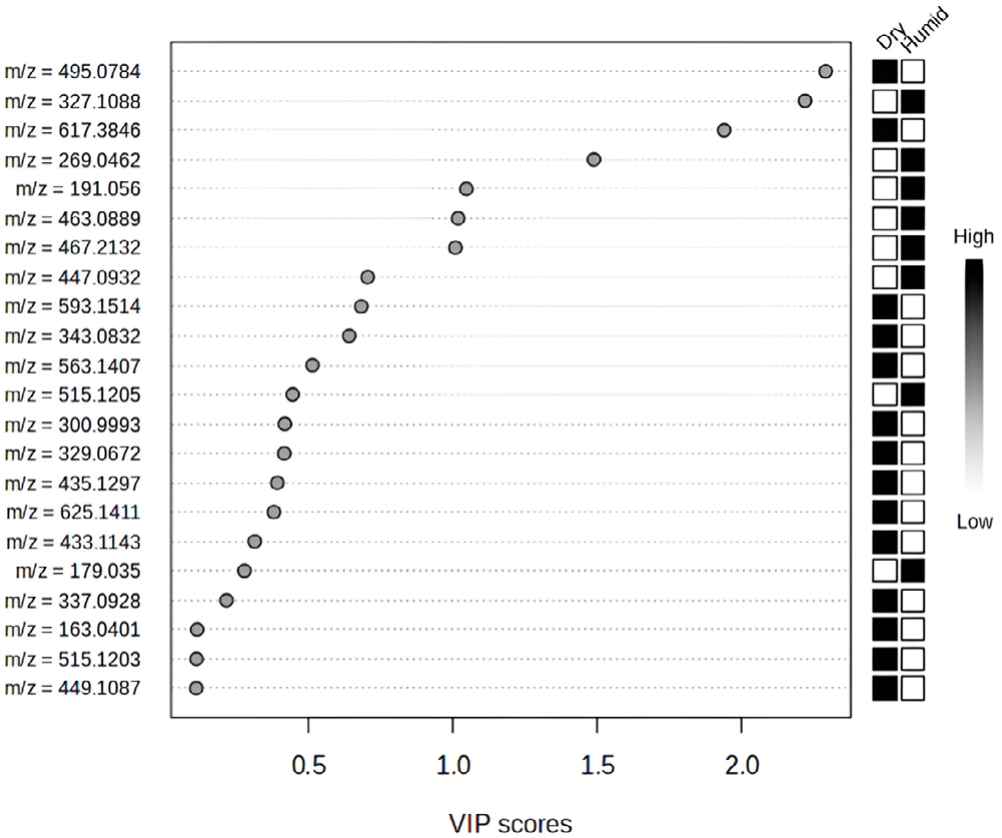
Variable Importance in Projection (VIP) scores from PLS‐DA based on UHPLC‐MS/MS data (negative ionization mode), showing the metabolites that best discriminate *Malpighiaceae* leaf samples from dry and humid tropical areas. Higher scores indicate greater contribution to group separation.

The VIP Score was applied to identify the metabolites associated with the observed discrimination. A total of 22 compounds were listed in the VIP Score analysis (Figure 5), with each metabolite assigned a Score based on its contribution to the metabolomic differentiation between leaves from dry and humid tropical areas. Table S2 provides detailed information on the identified metabolites, derived from UHPLC‐MS/MS profiles in negative ionization mode for Malpighiaceae species (Figure 5).

The highest VIP Score (negative ionization mode) was attributed to the phenolic compound 3,4‐di‐*O*‐galloylquinic acid (Compound 1—Table S2), which showed the greatest relative intensity in *B. coccolobifolia* leaves cultivated in the Cerrado biome. Thus, this compound was the most significant contributor to the discrimination between tropical environments. The second highest VIP Score was assigned to metabolite (3), also a phenolic compound, identified as 1‐(4‐hydroxyphenyl)‐3‐[(2R,3R,4S,5S,6R)‐3,4,5‐trihydroxy‐6‐(hydroxymethyl)oxan‐2‐yl]oxypropan‐1‐one. The molecular ion corresponding to this compound (Table S2) was detected for the first time in the leaves of *Niedenzuella poeppigiana*, *Heteropterys intermedia* and *Heteropterys leona*.

Unlike anthocyanins, whose concentrations typically increase in response to water stress, not all phenolic compounds exhibit higher levels in dry environments [5]. This is exemplified by metabolites (1) and (3), both highlighted in the VIP Score (Figure 5). Although water stress is known to activate biosynthetic pathways for phenolic compounds [5], the ecological or physiological reason why metabolite (1—Table S2) accumulates in dry areas while metabolite (3—Table S2) is more prevalent in humid areas remains unclear. Nevertheless, the findings suggest that phenolic compounds—particularly metabolites (1—Table S2) and (3—Table S2)—play a significant role in distinguishing Malpighiaceae leaves from different tropical environments. Future ecophysiological studies focusing on these compounds may help elucidate their specific roles in plant adaptation.

The 3,4‐di‐*O*‐galloylquinic acid is linked to the shikimate‐derived gallic acid/ellagic acid pathway, which contributes to reactive oxygen species (ROS) scavenging, membrane protection and stress‐signalling mechanisms under limited water availability. The differential accumulation of these compounds observed in our analyses supports the notion that water‐scarcity conditions modulate secondary metabolic fluxes toward phenolic biosynthesis. These findings provide mechanistic insight into the metabolic adjustments occurring under drought stress and highlight promising targets for future research focusing on pathway‐level regulation and stress‐adaptive metabolite production.

Chromatographic profiles obtained by UHPLC‐MS/MS in negative ionization mode also led to the annotation (Table S2) of nine previously unreported metabolites in the studied Malpighiaceae species: 3,4‐di‐*O*‐galloylquinic acid (1), quinic acid (2), 1‐(4‐hydroxyphenyl)‐3‐[(2R,3R,4S,5S,6R)‐3,4,5‐trihydroxy‐6‐(hydroxymethyl)oxan‐2‐yl]oxypropan‐1‐one (3), caffeic acid (4), 3,4‐di‐*O*‐caffeoylquinic acid (6), 2‐oct‐1‐en‐3‐yloxy‐6‐[(3,4,5‐trihydroxyoxan‐2‐yl)oxymethyl]oxane‐3,4,5‐triol (7), quercetin‐3,4‐*O*‐di‐β‐glucoside (8), 1,3,4‐trihydroxy‐5‐[(E)‐3‐(4‐hydroxyphenyl)prop‐2‐enoyl]oxycyclohexane‐1‐carboxylic acid (12) and luteolin‐2‐*O*‐glucoside (14).

## 4 Conclusion

This study provides unprecedented insights into the metabolic responses of Malpighiaceae species to seasonal water stress, revealing, through high‐resolution UHPLC‐MS/MS and robust multivariate chemometric analyses, significant variations in metabolite synthesis between dry and humid tropical environments. The identification of 15 metabolites, including cyanidin‐3‐*O*‐sambubioside and 3,4‐di‐*O*‐galloylquinic acid, as key discriminants represents a major analytical milestone, as these compounds are reported here for the first time in this botanical family.

By integrating UHPLC‐MS/MS chromatographic datasets with chemometric discrimination, this study establishes a comprehensive and high‐resolution analytical strategy capable of elucidating how contrasting environmental conditions modulate metabolic biosynthesis. This integrative framework enhances interpretability, improves analytical robustness and clearly highlights the methodological innovation achieved, positioning the work as a relevant contribution to analytical science. Moreover, the approach demonstrates strong potential for adaptation to broader metabolomic investigations, especially in complex and environmentally driven biological matrices.

By establishing a clear association between water availability and the biosynthesis of secondary metabolites, the research advances our understanding of plant adaptation strategies to abiotic stress. Beyond contributing to the phytochemical mapping of underexplored taxa, these findings provide a valuable framework for future ecological and physiological studies, as well as for biotechnological applications aimed at exploiting stress‐resilience mechanisms for agricultural improvement, natural product discovery and biodiversity conservation.

Future research should prioritize the in‐depth elucidation of the metabolic routes and regulatory mechanisms underlying the differential biosynthesis of secondary metabolites observed under contrasting water‐availability conditions. Integrating metabolomics with transcriptomic, proteomic and fluxomic approaches will be essential to determine how specific enzymatic pathways are activated, suppressed or reconfigured during seasonal water stress. Additionally, comparative analyses across other Malpighiaceae genera, as well as phylogenetically related families, could clarify whether the metabolic signatures identified here represent lineage‐specific adaptations or broader stress‐responsive strategies. Advancing these mechanistic and pathway‐oriented investigations will not only deepen our understanding of plant resilience to abiotic stress but also enhance the strategic exploitation of metabolic traits with ecological, agronomic and biotechnological relevance.

## Author Contributions

**Jaqueline Munise Guimarães da Silva**: conceptualization, formal analysis, investigation, methodology, resources, software, validation, writing – original draft, writing – review and editing. **Rafael Felipe de Almeida**: conceptualization, data curation, resources, validation, writing – original draft. **Maria Luiza Zeraik**: conceptualization, project administration, resources, supervision, validation, writing – original draft, writing – review and editing. All authors have read and agreed to the published version of the manuscript.

## Conflicts of Interest

The authors declare no conflicts of interest.

## Supporting information




**Supporting File**: ansa70061‐sup‐0001‐SuppMat.docx.

## Data Availability

The data that supports the findings of this study are available in the supplementary material of this article.
