# Supplementary material for: Metabolomic Differentiation of Malpighiaceae From Dry and Humid Tropics via UHPLC‐MS/MS and Chemometrics
Source: Anal Sci Adv. 2026 Mar 6;7(1):e70061. doi: 10.1002/ansa.70061 (PMC12965184; doi:10.1002/ansa.70061)
Supplement: Supplementary file 1 — Supporting File: ansa70061‐sup‐0001‐SuppMat.docx. [file ANSA-7-e70061-s001.docx]

**Supporting Information**

**Metabolomic Differentiation of Malpighiaceae from Dry and Humid Tropics via UHPLC-MS/MS and Chemometrics**

Jaqueline Munise Guimarães da Silva^a^, Rafael Felipe de Almeida^b^, Maria Luiza Zeraik^a^*

^a^Laboratory of Phytochemistry and Biomolecules, Department of Chemistry, State University of Londrina (UEL), 86051–990, Londrina, PR, Brazil.

^b^C.E.Moss Herbarium, School of Animal, Plant and Environment, University of Witwatersrand, Johannesburg, 2092, South Africa.

*Author for correspondence: zeraikml@uel.br *

*TABLES*

*Table SI – 1. Metabolites annotated in the profiles obtained by UHPLC-MS/MS (positive ionization mode) of Malpighiaceae species listed in the VIP Score (PLS-DA).*

*Table SI – 2. Metabolites annotated in the profiles obtained by UHPLC-MS/MS (negative ionization mode) of Malpighiaceae species listed in the VIP Score (PLS-DA).*

Table SI – 1. Metabolites annotated in the profiles obtained by UHPLC-MS/MS (positive ionization mode) of Malpighiaceae species listed in the VIP Score (PLS-DA).

| **N°** | **R_t_*** | **Molecular formula** | **Ion (*m*/*z*)**  **[M+H]^+^** | **Exact mass** | **Error**  **(ppm)** | **Fragment** | **Class** | **Metabolites** | **Tropical area** | **Leaves** |  |
| --- | --- | --- | --- | --- | --- | --- | --- | --- | --- | --- | --- |
| 1 | 0.27 | C_30_H_27_O_12_ | 579.1510 | 579.1503 | 1.20 | 409.0915 (40), 287.0567 (80), 275.0567 (60), 247.0606 (50), 233.0458 (30), 163.0411 (50), 139.0405 (70), 127.0385 (100), 123.0452 (40) | anthocyanin | proanthocyanidin | Dry | *N. multiglandulosa* |  |
| 2 | 2.53 | C_27_H_31_O_17_ | 627.1566 | 627.1561 | 0.79 | 301.0348 (100) | flavonoid | quercetin–3,4–*O*–di–glycoside | Dry | *N. multiglandulosa; B. coccolobifolia; D. pubipetala; B. laevifolia; B. malifolia* |  |
| 3 | 2.87 | C_30_H_25_O_12_ | 577.1340 | 577.1346 | –1.03 | 289.0719 (100) | anthocyanin | procyanidin | Dry | *D. pubipetala* |  |
| 4 | 2.90 | C_26_H_29_O_14_ | 565.1550 | 565.1557 | –1.23 | 325.1134 (100) | flavonoid | isovitexin–2–*O*–arabinoside** | Dry | *N. multiglandulosa* |  |
| 5 | 2.91 | C_27_H_31_O_14_ | 579.1699 | 579.1713 | –2.45 | 433.1145 (20), 415.1025 (15), 397.0899 (30), 379.0808 (45), 367.0820 (45), 349.0735 (15), 337.0713 (90), 313.0711 (100), 283.0600 (85) | flavonoid | vitexin–2–*O*–ramnoside** | Dry | *B. malifolia* |  |
| 6 | 2.93 | C_27_H_31_O_16_ | 611.1606 | 611.1612 | –0.98 | 303.0509 (100) | flavonoid | delfinidin–3–*O*–6–*O*–alpha–rhamnopyranosyl–beta–glucopyranoside** | Dry | *N. multiglandulosa; B. coccolobifolia; D. pubipetala; B. laevifolia; B. malifolia* |  |
| 7 | 2.96 | C_26_H_30_O_15_ | 582.1583 | 582.1584 | –0.17 | 287.0538 (100) | anthocyanin | cyanidin–3–*O*–sambubioside** | Dry | *N. multiglandulosa; B. coccolobifolia; D. pubipetala; B. laevifolia; B. malifolia* |  |
| 8 | 3.04 | C_27_H_31_O_15_ | 595.1652 | 595.1663 | –1.84 | 287.0555 (100) | flavonoid | kaempferol–3–*O*–rutinoside | Dry | *N. multiglandulosa; B. coccolobifolia; D. pubipetala; B. laevifolia; B. malifolia* |  |
| 9 | 3.12 | C_20_H_19_O_11_ | 435.0916 | 435.0927 | –2.52 | 303.0491 (100) | flavonoid | quercetin–3–*O*–xyloside | Dry | *B. coccolobifolia* |  |
| 10 | 3.13 | C_27_H_29_O_15_ | 593.1503 | 593.1506 | –0.50 | 285.0341 (100) | flavonoid | kaempferol–acetate–*O*–pentosyl–pentoside** | Humid | *H. leona; S. saxicola* |  |
| 11 | 3.14 | C_27_H_31_O_15_ | 595.1654 | 595.1663 | –1.51 | 541.1243 (20), 523.1271 (20), 481.1141 (40), 457.1110 (45), 427.1029 (65), 409.0926 (75), 379.0817 (90), 337.0733 (55), 325.0723 (100), 307.0594 (45), 295.0613 (30) | flavonoid | genistein–di–*C*–hexoside** | Dry | *N. multiglandulosa; D. pubipetala; B. laevifolia; B. malifolia* |  |
| 12 | 3.17 | C_27_H_31_O_16_ | 611.1611 | 611.1612 | –0.16 | 303.0514 (100) | flavonoid | rutin | Dry | *D. pubipetala; B. laevifolia; B. malifolia* |  |
| 13 | 3.20 | C_28_H_33_O_16_ | 625.1764 | 625.1768 | –0.47 | 317.0677 (100) | flavonoid | isorhamnetin–3–*O*–rutinoside | Dry | *B. laevifolia* |  |
| 14 | 3.43 | C_27_H_45_O_7_ | 481.3164 | 481.3165 | –0.20 | 481.1700 (10), 445.2974 (100), 427.2840 (55), 409.2766 (40), 371.2234 (70), 162.1287 (80) | steroid | ecdisterona | Dry | *B. laevifolia* |  |
| *R_t_: retention time (min); **metabolite first annotated in the leaves of Malpighiaceae species. | | | | | | | | | | | |

Table SI – 2. Metabolites annotated in the profiles obtained by UHPLC-MS/MS (negative ionization mode) of Malpighiaceae species listed in the VIP Score (PLS-DA).

| **N°** | **R_t_*** | **Molecular formula** | **Ion (*m*/*z*)**  **[M–H]^–^** | **Exact mass** | **Error**  **(ppm)** | **Fragnents** | **Class** | **Metabolites** | **Tropical area** | **Leaves** |  |
| --- | --- | --- | --- | --- | --- | --- | --- | --- | --- | --- | --- |
| 1 | 2.29 | C_21_H_19_O_14_ | 495.0784 | 495.0774 | 2.01 | 169.0137 (100) | phenolic compound | 3,4–di–*O*–galloylquinic acid** | Dry | *B. coccolobifolia* |  |
| 2 | 2.37 | C_7_H_11_O_6_ | 191.0560 | 191.0555 | 2.61 | 173.0455 (75), 111.3352 (100) | phenolic compound | quinic acid** | Humid | *S. saxicola* |  |
| 3 | 2.48 | C_15_H_19_O_8_ | 327.1088 | 327.1079 | 2.75 | 148.0530 (100) | phenolic compound | 1–(4–hydroxyphenyl)–3–(2R,3R,4S,5S,6R)–3,4,5–trihydroxy–6–(hydroxymethyl)oxan–2–yloxypropan–1–one** | Humid | *N. poeppigiana; H. intermedia; H. leona* |  |
| 4 | 2.84 | C_9_H_7_O_4_ | 179.0350 | 179.0344 | 3.35 | 135.0434 (100) | phenolic compound | caffeic acid** | Humid | *H. intermedia; S. saxicola* |  |
| 5 | 2.93 | C_14_H_5_O_8_ | 300.9993 | 300.9984 | 2.99 | 271.9027 (100) | phenolic compound | ellagic acid | Dry | *B. coccolobifolia; D. pubipetala* |  |
| 6 | 3.09 | C_25_H_23_O_12_ | 515.1205 | 515.1189 | 3.10 | 190.0480 (50), 174.0530 (100) | phenolic compound | 3,4–di–*O*–caffeoylquinic acid** | Humid | *H. intermedia* |  |
| 7 | 3.31 | C_20_H_35_O_12_ | 467.2132 | 467.2128 | 0.85 | 276.1572 (100) | glycoside | 2–oct–1–en–3–yloxy–6–[(3,4,5–tri–hydroxyoxan–2–yl)oxymethyl]oxane–3,4,5–triol** | Humid | *H. intermedia; S. saxicola* |  |
| 8 | 3.32 | C_27_H_29_O_17_ | 625.1411 | 625.1404 | 1.11 | 301.0350 (100) | flavonoid | quercetin–3,4'–*O*–di–beta–glucoside** | Humid | *H. leona; A. latifolia* |  |
| 9 | 3.66 | C_9_H_7_O_3_ | 163.0401 | 163.0395 | 3.68 | 145.0351 (100) | phenolic compound | coumaric acid | Dry | *B. malifolia* |  |
| 10 | 3.67 | C_26_H_27_O_14_ | 563.1407 | 563.1400 | 1.24 | 442.0910 (100), 413.0888 (10), | flavonoid | trihydroxyflavone–*C*–hexoside–*C*–pentoside | Dry | *D. pubipetala; B. malifolia* |  |
| 11 | 3.71 | C_15_H_9_O_5_ | 269.0462 | 269.0450 | 4.46 | 245.1394 (100) | flavonoid | apigenin | Dry | *N. multiglandulosa* |  |
| 12 | 3.73 | C_16_H_17_O_8_ | 337.0928 | 337.0923 | 1.48 | 161.0450 (100) | phenolic acid | (1R,3R,4S,5R)–1,3,4–trihydroxy–5–[(E)–3–(4–hydroxyphenyl)prop–2–enoyl]oxycyclohexane–1–carboxylic acid** | Humid | *H. intermedia; A. latifolia;* |  |
| 13 | 3.80 | C_21_H_19_O_11_ | 447.0932 | 447.0927 | 1.11 | 429.0739 (100), 403.1948 (40), 295.0689 (40), 277.0896 (60), 259.0403 (40) | flavonoid | luteolin–7–glicoside | Dry | *B. laevifolia* |  |
| 14 | 3.91 | C_21_H_23_O_10_ | 435.1297 | 435.1291 | 1.37 | 273.0794 (100) | flavonoid | phloretin–2–*O*–glycoside** | Humid | *A. latifólia* |  |
| 15 | 3.82 | C_25_H_23_O_12_ | 515.1203 | 515.1189 | 2.71 | 285.0400 (100) | phenolic compound | 3,4–di–*O*–caffeoylquinic acid | Dry | *B. malifolia* |  |
| 16 | 3.85 | C_27_H_29_O_15_ | 593.1514 | 593.1506 | 1.34 | 236.0684 (100) | flavonoid | kaempferol–3–*O*–rutinoside | Dry | *D. pubipetala; B. malifolia* |  |
| 17 | 3.94 | C_17_H_13_O7 | 329.0672 | 329.0661 | 3.34 | 269.0461 (100), 244.0383 (10) | flavonoid | tricin | Dry | *N. multiglangulosa* |  |
| 18 | 3.97 | C_21_H_21_O_10_ | 433.1143 | 433.1134 | 2.07 | 343.0096 (10), 300.0522 (100), 271.0639 (10) | flavonoid | naringenin–7–*O*–glycoside | Dry | *B. malifolia* |  |
| 19 | 4.02 | C_21_H_21_O_11_ | 449.1087 | 449.1083 | 0.89 | 216.9397 (100) | flavonoid | eriodictyol–7–*O*–glycoside | Dry | *D. pubipetala* |  |
| 20 | 4.09 | C_27_H_31_O_14_ | 579.1737 | 579.1713 | 4.14 | 271.0598 (100) | flavonoid | naringin | Dry | *B. malifolia* |  |
| 21 | 5.38 | C_18_H_15_O_7_ | 343.0832 | 343.0817 | 4.37 | 300.1152 (100), 271.1209 (10) | flavonoid | nevadensin | Dry | *B. malifolia* |  |
| 22 | 5.81 | C_39_H_53_O_6_ | 617.3846 | 617.3842 | 0.64 | 541.8320 (20), 483.2845 (75), 423.2466 (100), 313.1736 (50) | terpene | 3–O–coumaroylalphytolic acid | Dry | *B. coccolobifolia* |  |
| *R_t_: retention time (min); **metabolite first annotated in the leaves of Malpighiaceae species. | | | | | | | | | | | |
